# From the feces to the genome: a guideline for the isolation and preservation of *Strongyloides stercoralis* in the field for genetic and genomic analysis of individual worms

**DOI:** 10.1186/s13071-019-3748-5

**Published:** 2019-10-22

**Authors:** Siyu Zhou, Dorothee Harbecke, Adrian Streit

**Affiliations:** 1 School of Preclinical Medicine, Guangxi Medical University, Nanning, Guangxi China; 2 Department of Integrative Evolutionary Biology, Max Planck Institute for Developmental Biology, Tübingen, Baden-Württemberg Germany

**Keywords:** Strongyloidiasis, *Strongyloides stercoralis*, Phylogeny, Single worm whole genome sequencing

## Abstract

Strongyloidiasis is a soil-borne helminthiasis, which, in spite of the up to 370 million people currently estimated to be infected with its causing agent, the nematode *Strongyloides stercoralis*, is frequently overlooked. Recent molecular taxonomic studies conducted in Southeast Asia and Australia, showed that dogs can carry the same genotypes of *S. stercoralis* that also infect humans, in addition to a presumably dog-specific *Strongyloides* species. This suggests a potential for zoonotic transmission of *S. stercoralis* from dogs to humans. Although natural *S. stercoralis* infections have not been reported in any host other than humans, non-human primates and dogs, other as yet unidentified animal reservoirs cannot be excluded. Molecular studies also showed that humans carry rather different genotypes of *S. stercoralis*. As a result, their taxonomic status and the question of whether they differ in their pathogenic potential remains open. It would therefore be very important to obtain molecular genetic/genomic information about *S. stercoralis* populations from around the world. One way of achieving this (with little additional sampling effort) would be that people encountering *S. stercoralis* in the process of their diagnostic work preserve some specimens for molecular analysis. Here we provide a guideline for the isolation, preservation, genotyping at the nuclear *18S* rDNA and the mitochondrial *cox*1 loci, and for whole genome sequencing of single *S. stercoralis* worms. Since in many cases the full analysis is not possible or desired at the place and time where *S. stercoralis* are found, we emphasize when and how samples can be preserved, stored and shipped for later analysis. We hope this will benefit and encourage researchers conducting field studies or diagnostics to collect and preserve *S. stercoralis* for molecular genetic/genomic analyses and either analyze them themselves or make them available to others for further analysis.
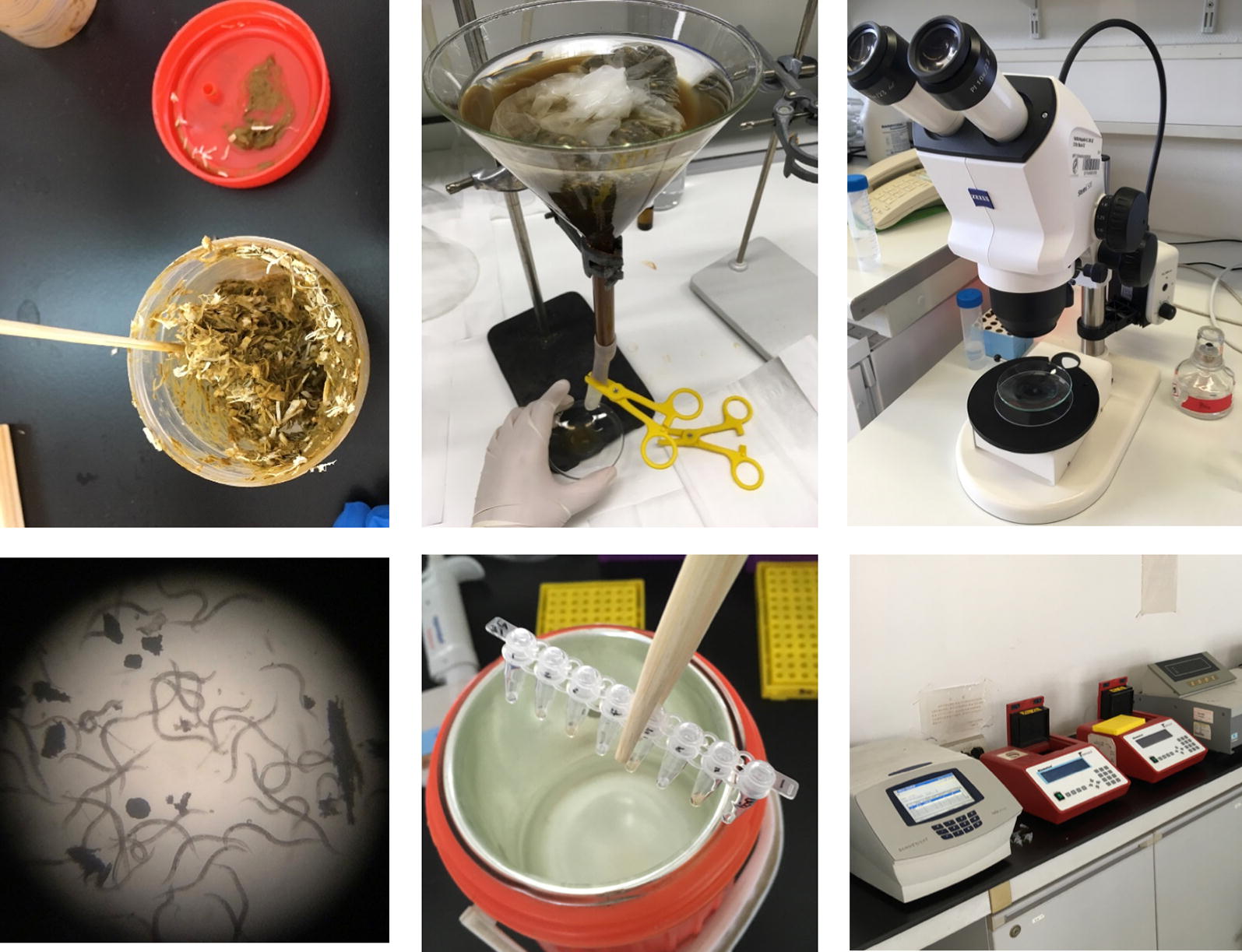

## Background

Strongyloidiasis is a soil-transmitted helminthiasis (STH) and although as such it is included within the neglected tropical diseases (NTDs), it is often overlooked in comparison with other STHs and has therefore sometimes been described as (one of) the most neglected NTDs [1–3]. Published estimates of the number of people currently infected with *Strongyloides stercoralis*, the species which causes the vast majority of human strongyloidiasis cases, vary between “30–100 million” [4, 5], and “at least 370 million” [2]. Given the difficulties with diagnosis, [5–8] the actual number may be even higher. In general, *S. stercoralis* is more prevalent in tropical and subtropical regions, with a local prevalence of 10–40% reported in some endemic areas [9, 10]. High temperature and moisture, poverty, poor sanitary conditions such as walking barefoot and open defecation are risk factors for *S. stercoralis* transmission [1]. However, strongyloidiasis is not limited to the tropical and subtropical regions and increasing numbers of *S. stercoralis* cases in areas with a moderate climate and comparably good sanitary conditions such as Europe, North America and Australia have been reported over the last years [11–16].

For various reasons, notably the absence of eggs from the stool and the often very low parasite burden, *S. stercoralis* remains frequently undetected in standard parasitological surveys [6]. However, since there is an increasing awareness of *S. stercoralis* and the difficulties with its diagnosis, increasing numbers of studies with specific methodology have been conducted [5–8]. Although PCR based methods are available [17, 18], in most cases *S. stercoralis* diagnostics rely upon the direct observation of worms in stool using techniques such as stool smears, Kato-Katz, formalin ethyl acetate concentration, agar plate culture, Harada-Mori filter paper culture and the Baermann technique. Agar culture and Baermann were found to be fairly highly sensitive, when multiple stool samples from consecutive days are examined ([1, 5] and references therein).

Although the biology and pathology of *S. stercoralis* has been studied for more than 100 years [19], many questions remain open. An important one is to what extent strongyloidiasis is a zoonotic disease. In particular, dogs have been implicated as a possible animal reservoir; Brumpt [20] had proposed a species named *S. canis* as the natural *Strongyloides* species in dogs, which is different from *S. stercoralis* found in human and non-human primates. Although this was originally supported by some authors [21, 22], later work [23] considered *S. canis* a *nomen dubium*, mainly due to the lack of a proper description. Given that no morphological differences could be observed between *S. canis* and *S. stercoralis* and that dogs are clearly suitable experimental hosts for human-derived *S. stercoralis* [24, 25], the existence of a separate species *S. canis* was not generally accepted. Hence, it remained open whether *Strongyloides* spp. found naturally in humans and in dogs are actually the same species or not. Recent molecular taxonomic studies conducted in Southeast Asia [26, 27] and Australia [18] showed that dogs carry at least two different populations (probably species) of *Strongyloides*, one of which appears to be the same *S. stercoralis* that also infects humans, underpinning the potential for zoonotic *S. stercoralis* transmission from dogs to humans. No comparable studies from other parts of the world are available. Interestingly, the very few *Strongyloides* worms isolated from dogs elsewhere in the world for which DNA sequence information was reported, appear to be of the human-infective type [28–32]. Although natural *S. stercoralis* infections have not been consistently reported in any host other than humans, non-human primates and dogs, other as yet unidentified animal reservoirs cannot be excluded. Based on morphology, *Strongyloides* larvae or free-living adults can normally be reliably identified to the genus (*Strongyloides*) but the species is frequently inferred from the host because of the lack of conclusive morphological differences, particularly in the developmental stages existing outside of the host [33, 34]. Further, as humans carry rather different *18S* rDNA and *cox*1 genotypes, it remains unclear what their exact phylogenetic relationship is and if they might differ in their pathogenic potential [26, 27, 35].

To clarify these issues, it would be most valuable to obtain molecular genetic/genomic information about *S. stercoralis* populations from around the world. One way of achieving this with little additional sampling effort would be that people encountering *S. stercoralis* in the process of their diagnostic work preserve some specimens for molecular analysis.

In this paper we provide a guideline for the genotyping and whole genome sequencing of single *S. stercoralis*, from worm isolation in the field, to the generation of the sequences in the laboratory (the bioinformatics analysis of the sequences is not part of this article). Because in many cases, a lack of equipment, time, funds or expertise may exist, or the full analysis is not possible, necessary or desired at the place and time where *S. stercoralis* are found, we emphasize here when and how samples can be preserved and shipped for later analysis. We hope this will benefit and encourage researchers conducting field studies or diagnostics to collect and preserve *S. stercoralis* for molecular genetic/genomic analyses and either analyze them themselves or send them to an appropriately equipped laboratory for further analysis.

## Part 1. In the field: collection and preservation

**Fig. 1:**
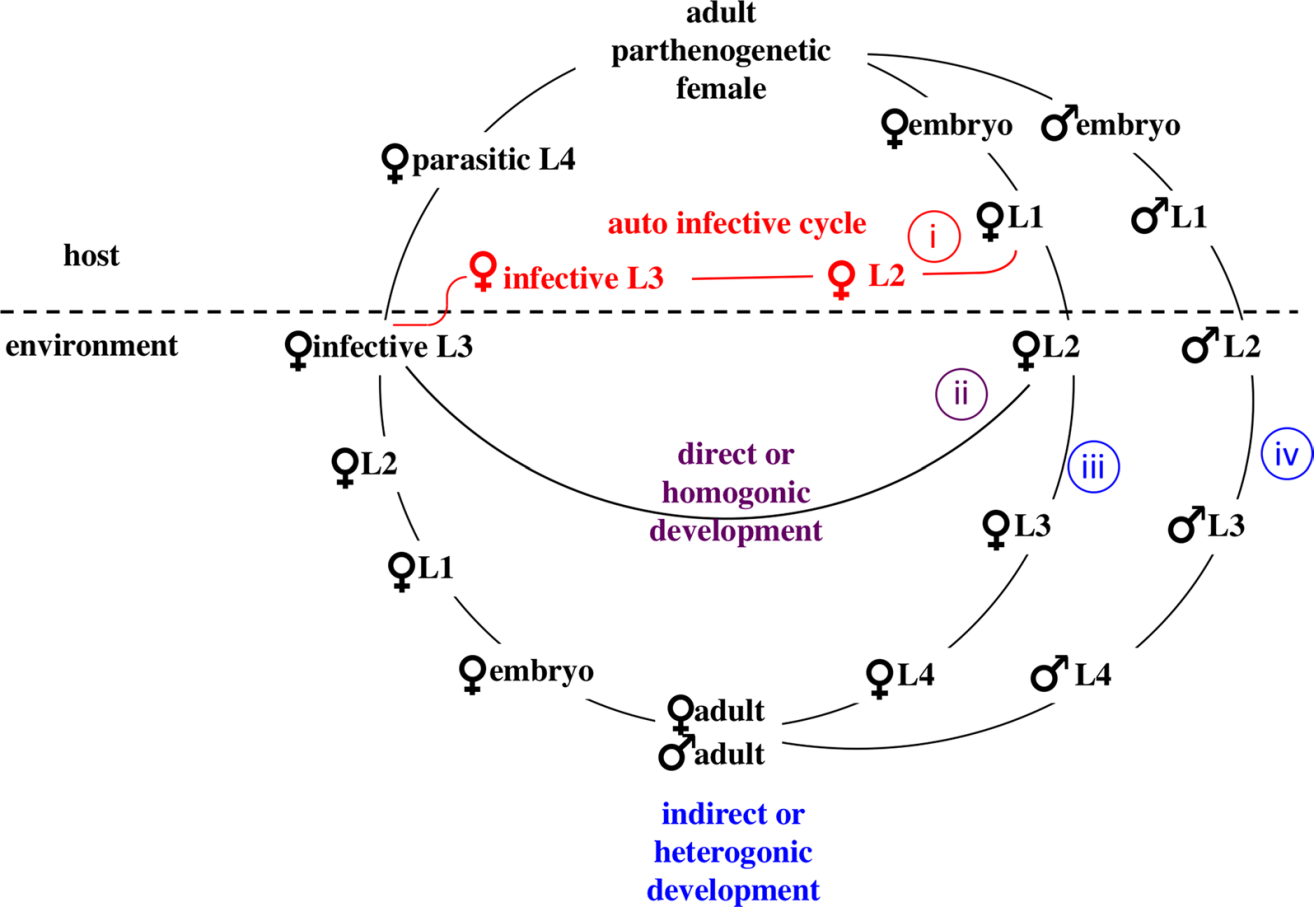
The life-cycle of *S. stercoralis.* Infective third-stage larvae (iL3s), which are all females, invade a new host by skin penetration and eventually establish in the small intestine of the host. The parasitic adult females reproduce by parthenogenesis and their progeny have four developmental options: (i) they may become female, and develop into infective third-stage larvae (iL3) within the host and re-infect the same host individual (autoinfective cycle); (ii) they may become female, leave the host as first-stage larvae and develop into iL3 in the environment and search for a new host (direct/homogonic development); (iii) they may become female, leave the host as first-stage larvae, and develop into free-living, non-infective third-stage larvae and subsequently into adult females (indirect/heterogonic development); (iv) they may become male and develop into free-living adult males (indirect/heterogonic cycle). The free-living adults mate and reproduce sexually in the environment and all their progeny are females and develop to iL3s. Copyright: Creative Commons Attribution 4.0 License (https://creativecommons.org/licenses/by/4.0/). Modified from the original picture by A. Streit. Citation: Jaleta et al. (2017) Different but overlapping populations of *Strongyloides stercoralis* in dogs and humans-dogs as a possible source for zoonotic strongyloidiasis. *PLoS Neglected Tropical Diseases* 2017, 11(8):e0005752 [26]

In this first part we describe how *S. stercoralis* worms can be isolated, preserved and shipped. We describe our preferred method for *S. stercoralis* isolation, which includes culturing samples for 1–2 days followed by harvesting worms using the Baermann technique. This has the advantage that it allows the *S. stercoralis* worms to develop into infective larvae (homogonic development) or free-living females or males (heterogonic development), and therefore provides information on the sex and the developmental route that the analyzed worm underwent (Fig. 1). In the case of adults, it also leads to more DNA per worm compared with young larvae. However, we do know that this method is frequently not practical in large-scale routine diagnostics or parasitological surveys. Therefore, we would like to stress that worms of any developmental stage isolated by any method can be used for molecular analysis.

### Section 1: Fecal sampling from humans and animals

Soil nematodes are omnipresent and colonize deposited feces very quickly. While this causes no major problems for diagnostics using egg flotation, free-living nematodes are a disturbance in parasitic nematode cultures. Therefore, whenever possible, feces should be collected directly into sampling containers without contacting soil. This is usually the case for human samples. For animal samples, we suggest collecting feces directly from the rectum if possible, or to keep the animals on a clean surface and collect the fresh feces shortly after defecation.

### Section 2: Sample culturing and worm isolation

**Fig. 2:**
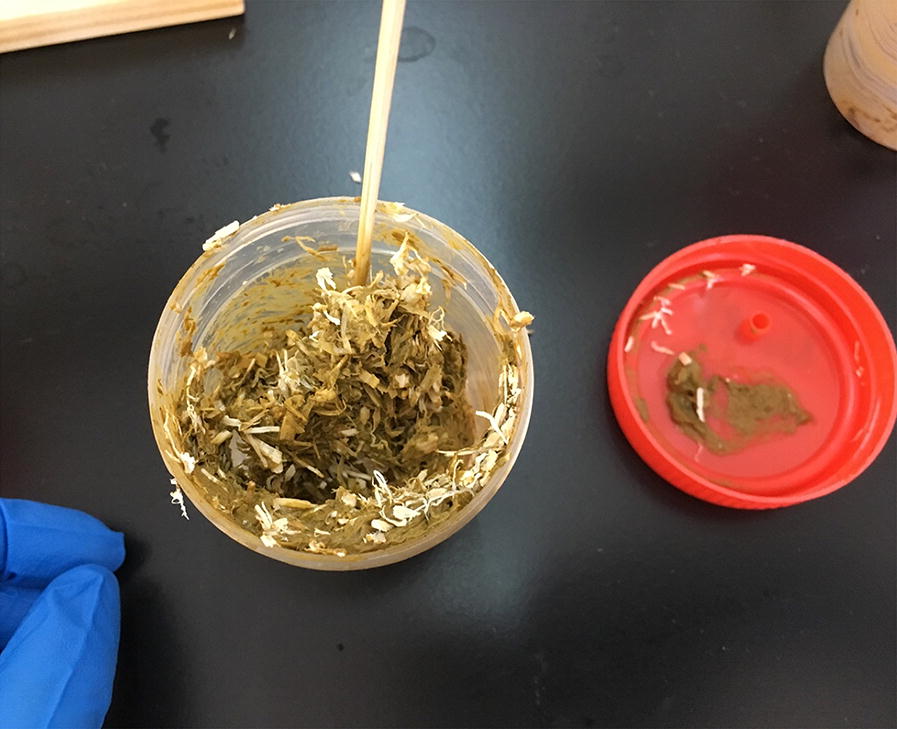
Fecal culture preparation. Mix the feces with an about equal amount of sterile saw dust (shown) or charcoal
(ii) Incubation conditions: Incubate the samples at 28 °C. If no incubator is available, samples can be kept at ambient temperature. *Strongyloides stercoralis* appears to be fairly temperature tolerant, as might be expected given that most *S. stercoralis* endemic regions have a tropical or subtropical climate. To maintain humidity, place some water in an open container in the incubator or cover the samples with a wet towel (Fig. 3). Do not refrigerate the samples or the cultures at any point. In our hands this is highly detrimental to the survival of various species of *Strongyloides*.

(i) Set up cultures: If very young larvae should be isolated, the feces can be directly processed without culturing [see (iii)]. Otherwise, mix the fresh feces with an about equal volume of sawdust or charcoal bits in a clean container or bag. If the feces are too dry, add some water. The cultures should form a loose, moist, crumbly mass and not be soaking wet (Fig. 2). This serves to allow gas exchange and prevent anaerobic conditions. To avoid hypoxia, do not seal the container or bag but leave a gap to allow air exchange. If possible, use about 50–100 g of feces for one culture; however, we have successfully isolated *S. stercoralis* from much less (only a few grams) feces.

**Fig. 3:**
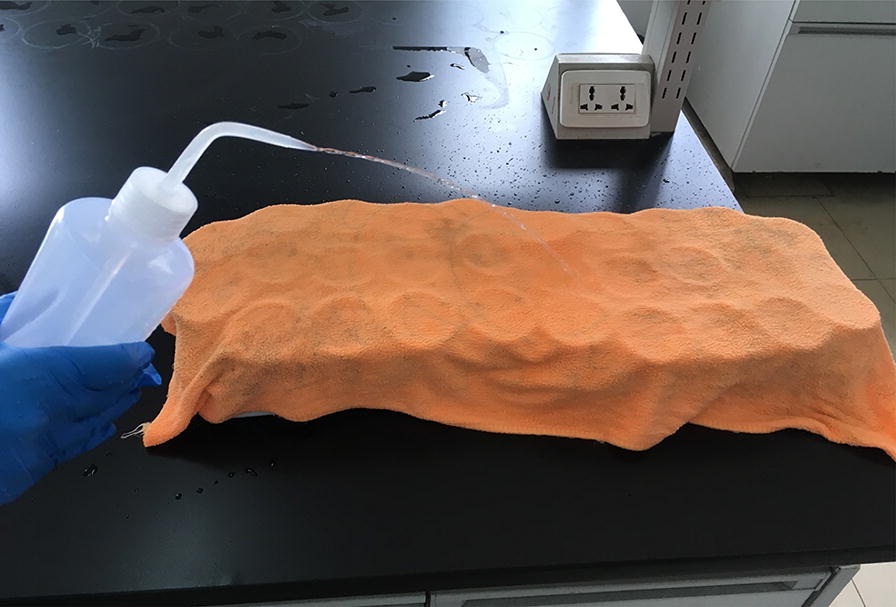
Fecal cultures covered with a wet towel
(iii) Incubation time: *S. stercoralis* larvae hatch within the host. Therefore, to collect young larvae, fresh fecal samples can be processed either directly or after being cultured for a few hours. To collect free-living adults and differentiated infective larvae (iL3s) of the direct (homogonic) cycle, samples should be cultured for 1–2 days. To collect iL3s of the indirect (heterogonic) cycle, samples should be cultured for 6–9 days.
(iv) Set up Baermann funnels: The simplest way is to place a piece of rubber tubing at the bottom of a glass funnel, close the end of the tube with a clamp and fill the funnel with tap water. Wrap the fecal sample with tissue paper and place it into the funnel (Fig. 4a). This can be done at ambient temperature. For a more sophisticated set up, see [25].

**Fig. 4:**
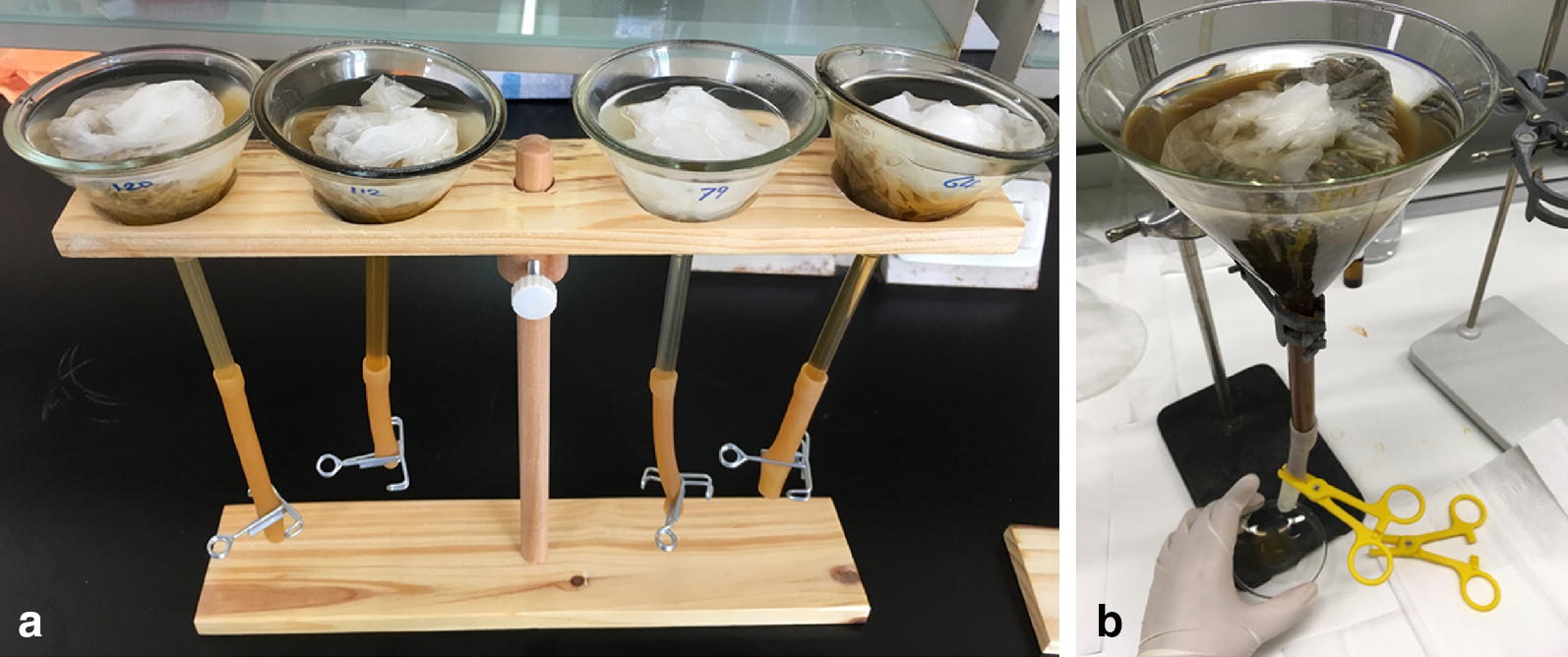
**a** Baermann funnel set up. **b** Worm harvest from the Baermann funnel
(v) Harvest worms: After the worms have crawled out of the feces and accumulated at the bottom of the funnel (allow at least two hours), place a second clamp 1–2 cm above the first clamp, remove the lower clamp and collect the worms into a watch glass or small Petri dish (Fig. 4b).

We recommend to transfer the worms into clean water and wash a few times prior to lysis or preservation in order to minimize the DNA derived from other organisms present in the fecal sample.

### Section 3: Morphological observation

*Strongyloides stercoralis* in the watch glass can be directly observed under a dissecting stereomicroscope with illumination from below. Depending on the incubation time and temperature, different developmental stages of worms can be observed.

The L1 and L2 larvae can be observed from fresh feces and after a few hours of culture. They are approximately 0.18–0.54 mm in length. For non-specialists they are the most difficult stages to tell apart from, e.g. young hookworm larvae. However, *S. stercoralis* is normally the only nematode species of which larvae rather than eggs can be found in fresh human stool samples.

**Fig. 5:**
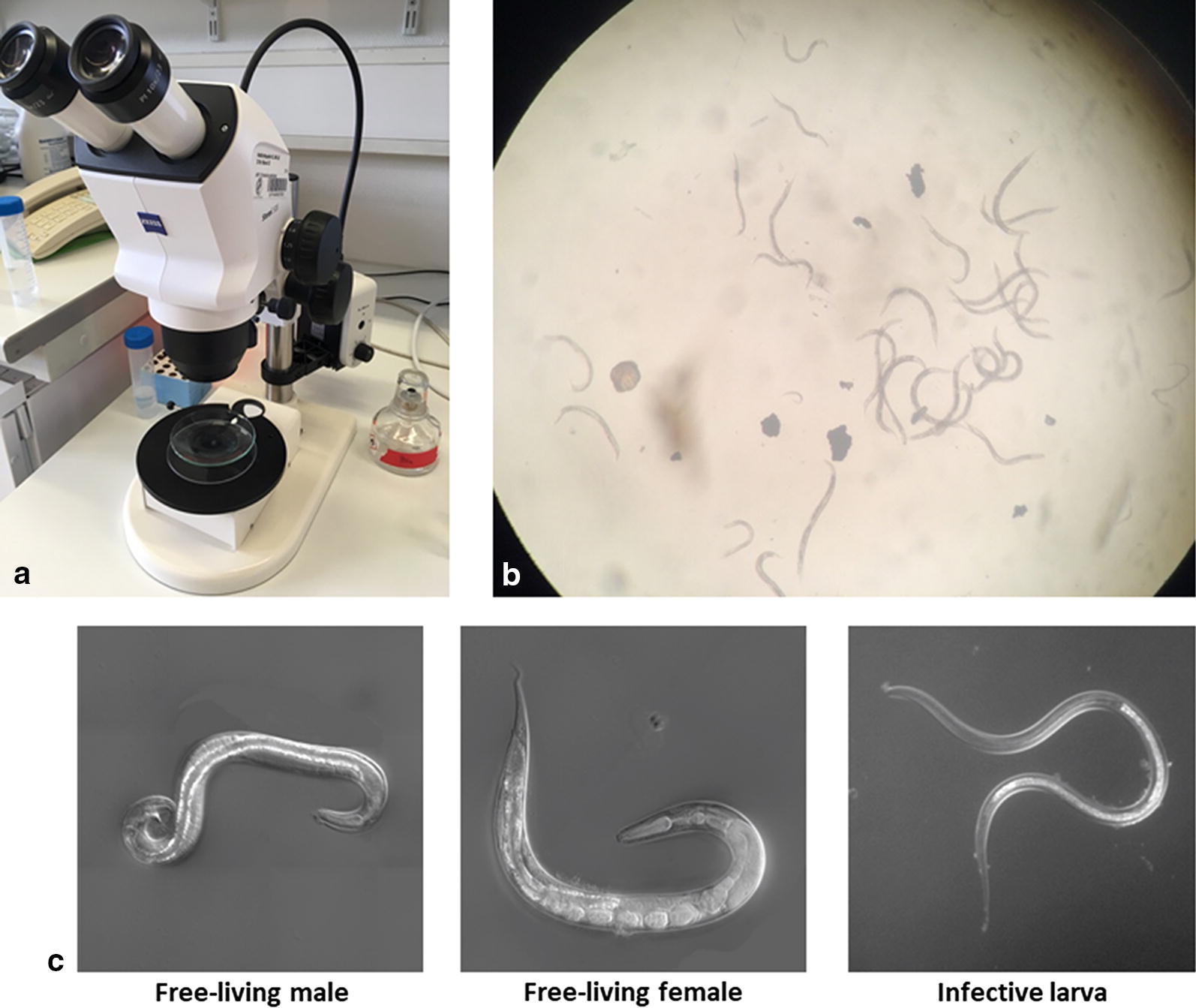
**a** Stereomicroscopic observation set up. It is important that the illumination is from underneath. **b** Free-living adults of *S. stercoralis* under the stereo dissecting microscopy at low magnification. **c** Morphology of free-living adults and iL3 of *Strongyloides* (shown are differential interference contrast (DIC) images of *S. papillosus*)

Free-living adults and differentiated infective iL3s of the direct (homogonic) cycle can be observed after one to two days of culture while the differentiated iL3s of the indirect (heterogonic) cycle can be observed after roughly 6 days (depending on the temperature). *Strongyloides* spp. are the only human gastrointestinal nematodes we are aware of that form reproductive adults outside of the host. The free-living adults are rhabditiform, approximately 0.8–1.2 mm in length and 40–70 μm in width. Females and males can be easily distinguished by their morphology (Fig. 5): Females are about 1.1–1.3 times larger than males. The female has a didelphic ovary and the vulva opens at mid-body length. The male has one testis, equal spicules and a ventrally curved tail.

The iL3s are filariform, approximately 0.4–0.8 mm in length and 14–19 μm in width and have a trifurcated tail. The most unique feature which sets *Strongyloides* (and *Parastrongyloides*) spp. apart from all other nematodes, is their clear oesophagus which extends over almost half of the body length followed by the much darker intestine.

Since we assume that most readers are accomplished diagnosticians who can recognize *S. stercoralis*, we do not provide further instructions on how to identify *S. stercoralis* based on their morphology. For a detailed morphological description of *S. stercoralis* we refer the reader to the classical paper [36] (reproduced in [37]).

### Section 4: Sample preservation

After the steps described above, the *S. stercoralis* worms are alive in clean water. They can be kept in this stage at ambient temperature (in our hands tested from 25 °C to 32 °C) for several hours or in the case of iL3s several days. Consequentially, iL3s can be shipped in water by express carrier in cases where shipment of live infective material is admissible. Below we describe three procedures for sample preservation: (A) individual worms frozen in water; (B) individual worms stored in ethanol at ambient temperature; and (C) pools of worms stored in ethanol at ambient temperature.

Use (A) if: (i) you have the time and opportunity to isolate single worms right away; (ii) you have the opportunity to keep (and, if required, ship) the samples frozen until DNA extraction.

Use (B) if: (i) you have the time and opportunity to isolate single worms right away; (ii) you do not have the opportunity to keep (and, if required, ship) the samples frozen until DNA extraction.

Use (C) if: you want to store the worms as a batch and single out individuals later or process them as a batch.

#### A. Preservation of individual worms in water (frozen)

For each worm:

(i) Add 9 μl of water into a PCR tube.
(ii) Transfer an individual worm in about 1 μl of water into the PCR tube using a mouth pipette or a micropipette (alternatively, use a worm pick to transfer the worm into a PCR tube containing 10 μl of water).
(iii) Store frozen until analysis, preferably at −80 °C or lower, but −20 °C will do for a number of years (the oldest sample we tested had been stored for three years at −20 °C).
(iv) If shipping is required, samples should be kept frozen (e.g. on dry ice).

#### B. Preservation of individual worms in ethanol (ambient temperature)

For each worm:

(i) Add 9 μl of water into a PCR tube.
(ii) Transfer an individual worm in about 1 μl of water into the PCR tube as described in (A).
(iii) Add 100 μl of 96–100% ethanol.
(iv) Seal the lid with parafilm to avoid ethanol evaporation.
(v) Store and ship the samples at ambient temperature.

#### C. Preservation of a pool of worms in ethanol (ambient temperature)

For each pool:

(i) Transfer the pool of worms in as little water as possible into an appropriately sized closable tube.
(ii) Keep the tube vertical and let the worms settle down. After 10 min, remove the supernatant as much as possible and estimate the volume.
(iii) Add 10 times the volume of 100% ethanol.
(iv) Close the tube and seal with parafilm to avoid ethanol evaporation.
(v) Store and ship the samples at ambient temperature.

## Part 2. In the laboratory: molecular analysis

Below we describe our preferred method for DNA extraction from the single worms. While undoubtedly many other DNA extraction methods would also work, this method is rather simple, fast, does not require any commercial kits and is suitable for processing samples on a large scale. This method can also be used for extracting DNA from pools of worms. We also describe the protocol for PCR amplification and sequencing of genomic loci and we recommend several loci for *Strongyloides* species identification. Additionally, we provide a relatively simple and cost-effective solution for the whole genomic library preparation of single worms.

### Section 1: DNA extraction from single worms

#### Composition of 2× lysis buffer

20 mM Tris-HCl (pH = 8.3), 100 mM KCl, 5 mM MgCl_2_, 0.9% NP-40, 0.9% Tween 20, 240 μg/ml Proteinase K (Proteinase K should be stored at −20 °C, and freshly added to the lysis buffer shortly before use).

#### A. For worms preserved individually in water

(i) Freeze the sample by immersing the tube in liquid Nitrogen for a few seconds (or in a freezer at −80 °C for a few minutes) and thaw at room temperature; vortex and briefly centrifuge. Repeat the freeze-thaw cycle 3 times.
(ii) Add 10 μl 2× lysis buffer and mix.
(iii) Incubate at 65 °C for 2 h. Remark: at this step several published protocols, including some from our laboratory, call for inactivation of the proteinase K at 95 °C. We found this step to be unnecessary for subsequent PCR and even highly detrimental for the genomic library preparation using the tagmentation technique described in this paper (see Section 3 below), presumably due to partial denaturation of the DNA caused by the high temperature.
(iv) The lysate can be used for PCR immediately or stored at −20 °C. This is also a stage at which the samples can be shipped. Preferentially, they should be kept frozen but the DNA is fairly stable and we have shipped successfully on wet ice.

#### B. For worms preserved individually in ethanol

(i) With a pipette, remove as much of the ethanol as possible without discarding the worm.
(ii) Let the remaining liquid evaporate by leaving the sample with the lid open. Alternatively, the evaporation can be accelerated by using a vacuum concentrator.
(iii) Add 10 μl water.
(iv) Perform the lysis as described above in (A).

#### C. For a pool of worms preserved in ethanol

(i) Transfer the worms with ethanol into a watch glass.
(ii) Remove as much of the liquid as possible without discarding the worms.
(iii) To rehydrate the worms, add water into the watch glass. At first, the worms will float on the water surface. Remove as much of the liquid as possible without discarding worms, and add water again. Repeat this washing with water step at least two more times.
(iv) Add 9 μl water in a PCR tube.
(v) Transfer individual worms into the PCR tube (one worm/tube) with a micropipette or using a worm pick.
(vi) Perform the lysis as described above in (A).

### Section 2: PCR amplification and sequencing of selected loci

As a basis for a first molecular classification of the *Strongyloides* species, we recommend to PCR amplify and sequence the nuclear *18S* rDNA (*SSU*) hypervariable regions (HVR) I and IV and a portion of the mitochondrial gene *cox*1, as suggested by Hasegawa and colleagues [29, 30, 38]. Additionally, the worm lysates are also suitable for the amplification of single copy loci. As a well-working example, we describe here *ytP274* (a polymorphic single copy locus) that was used by [26] to demonstrate sexual reproduction and can serve as a positive control.

The lysates of single worms can be directly used for PCR amplification. Normally 1–2 µl lysate is sufficient for amplification. Here we describe our PCR protocol using *Taq* DNA polymerase and ThermoPol Reaction Buffer (M0267S, New England BioLabs, Ipswich, USA). Other polymerases also work but the protocol may need minor modifications according to the instructions of the respective manufacturer (e.g. 72 °C instead of 68 °C for the extension step). The preparation of a 25 µl PCR reaction mix is described in Table 1. The primers and their respective annealing temperatures are listed in Table 2. The PCR cycling program is as follows: an initial denaturation step at 95 °C for 30 s, followed by 35 cycles of (denaturation at 95 °C for 20 s, annealing for 15 s, extension at 68 °C for 90 s), and a final extension step of 5 min at 68 °C.

**Table 1:** PCR reaction using single worm lysate

Component	Amount
Single worm lysate	2.0 µl
Forward primer (10 μM)	0.5 µl
Reverse primer (10 μM)	0.5 µl
dNTPs (10 mM each)	0.4 µl
*Taq* DNA polymerase	0.3 µl (1.5 units)
10× ThermoPol Reaction Buffer	2.5 µl
H_2_O	18.8 µl

**Table 2:** Primers, annealing temperatures and product size

Region amplified	Primer		Sequence (5′–3′)	Annealing T (°C)	Product size (bp)	Reference
*18S* HVR-I	Forward	ZS6492; SSU18A^a^	AAACATGAAACCGCGGAAAG; AAAGATTAAGCCATGCATG	52	825 (ZS6492 and SSU26R); 862 (SSU18A and SSU26R)	[41]
	Reverse	SSU26R^a^	CATTCTTGGCAAATGCTTTCG			
	Sequencing	SSU9R^a^	AGCTGGAATTACCGCGGCTG			[40]
*18S* HVR-IV	Forward	18SP4F	GCGAAAGCATTTGCCAA	57	712	[29]
	Reverse	18SPCR	ACGGCCGGTGTGTAC			
	Sequencing	ZS6269	GTGGTGCATGGCCGTTC			[35]
*ytP* 274	Forward	TJ6026	CAGGACCACCTGGACAAGTT	54	543	[26]
	Reverse	TJ6027	CTTTCCATCCTGATGCCACT			
	Sequencing	TJ6026	CAGGACCACCTGGACAAGTT			

^a^In some publications, SSU18A is referred to as SSUA (e.g.[40, 43]); in publications from our department, SSU18A, SSU26R and SSU9R are also referred to as RH5401, RH5402 and RH5403, respectively [35, 44]

*Abbreviation*: T, temperature

The PCR product (0.5–1 µl) can be directly used for conventional Sanger sequencing with the primers listed in Table 2 using the BigDye Terminator v3.1 Cycle sequencing Kit (Applied Biosystems, Foster City, USA). Using other kits or submitting to a commercial sequencing facility might require additional purification of the PCR product.

For the *SSU* HVR-I, many studies in various nematodes used the primer pair SSU18A and SSU26R, because this primer pair is fairly universal and can also amplify the *SSU* HVR I from other nematodes such as hookworms [26, 35, 39–42]. However, SSU18A is highly AT rich (37% GC) with a low Tm (49 °C) and does not match the *S. stercoralis* sequence perfectly. In our hands, PCR amplification from single worms, especially larvae, with this primer failed relatively frequently. Therefore, in cases where a worm is already known to be *Strongyloides* species, we recommend the use of ZS6492 as a forward primer. In our hands this primer works more reliably, compared with SSU18A, for *Strongyloides* spp. but does not work well for hookworms and presumably other nematodes.

### Section 3: Whole genome sequencing of single worms

The worm lysate of single worms (as described above in Section 1) can be used to generate DNA libraries for whole genome sequencing. The methods for library preparation with very little DNA as input are currently evolving very rapidly and we recommend checking for improved methodology before starting with whole genome sequencing.

We have used several commercial low input kits, such as the Nextera DNA Library Prep Kit (Illumina, Dan Diego, USA), and the Low Input Library Prep Kit (Clontech, Kusatsu, Japan) [26]. Recently, we have developed a method based on Tn5 transposome tagmentation [45, 46], either with free Tn5 or with bead immobilized Tn5 [35].

Below we describe a protocol, which worked for *S. stercoralis* and certain other nematodes. We use the Tn5 transposomes from the Illumina Nextera DNA Library Prep kit. The Tn5 (170 µl) of one kit is sufficient for the preparation of about 8500 single worm libraries of *S. stercoralis* making it cost effective. The required reagents including the Tn5 transposomes can also be prepared in the laboratory [46] (the Tn5 preparation is not part of this paper).

#### Reagents needed

(i) Tn5 transposomes: TDE1 (Tagment DNA Enzyme) (catalog no. 15027865, Illumina), stored at −20 °C.
(ii) SpeedBeads^TM^: from GElifesciences (GE65152105050250, Chicago, USA), also available through Sigma-Aldrich (GE65152105050250, St. Louis, USA) stored at 4 °C.
(iii) Bead buffer: 18% PEG8000, 2.5 M NaCl, 10 mM Tris-HCl (pH = 8.0), 1 mM EDTA (pH = 8.0), stored at 4 °C.
(iv) 5× TAPS-DMF MgCl_2_ buffer: 50 mM TAPS (C_7_H_17_NO_6_S), 25 mM MgCl_2_, 50% DMF (N,N-dimethylformamide), stored at −20 °C.
(v) Dialysis buffer: 100 mM HEPES-KOH (pH = 7.2), 0.2 M NaCl, 0.2 mM EDTA, 0.2% Triton X-100, 20% glycerol, stored at 4 °C.
(vi) Primers: Illumina Nextera primers i5 and i7, stored at −20 °C. The sequences of the primers are available from Illumina (https://support.illumina.com/downloads/illumina-adapter-sequences-document-1000000002694.html) and can also be ordered from other suppliers.
(vii) Q5 High-Fidelity DNA polymerase and Q5 Reaction buffer: from New England BioLabs (M0491S, Ipswich, USA), stored at −20 °C.

#### Procedure

(i) Bead preparation: Transfer 1 ml SpeedBeads^TM^ into a 1.5 ml Eppendorf tube and incubate for 1 min on a magnetic stand. Remove the supernatant and wash the beads with 1 ml 10 mM Tris-HCl (pH = 8.0). Wait until the liquid is clear, remove the supernatant and resuspend beads in 50 ml bead buffer.
(ii) DNA clean-up with beads: Mix 20 µl of worm lysate (adjust with water if the lysate is less than 20 µl) with 4 µl of beads and 16 µl bead buffer. Incubate the mixture for 10 min at RT and then for 5 min on a magnetic stand. Remove the supernatant and wash 2 times with 200 µl 80% ethanol while keeping the tube on a magnetic stand. Remove the ethanol by pipetting and let beads and dry for a few minutes. Remove from the stand and resuspend beads in 7 µl of 10 mM Tris-HCl (pH = 8.0) and incubate for 5 min. Place the tube back on the magnetic stand and incubate for 5 min. Transfer the 7 µl supernatant (DNA) to a new tube without disturbing the beads.
(iii) DNA tagmentation: Mix the 7 µl of DNA from (ii) with 2 µl H_2_O, 2 µl 5× TAPS-DMF buffer, and 1 µl Tn5 (1:50 diluted in dialysis buffer). Incubate the mixture at 55 °C for 14 min and then cool it to 10 °C. In this step the Tn5 dilution is crucial, as excessive or insufficient tagmentation would lead to the failure of library preparation. Should another input DNA (e.g. different nematode species, pools of worms) or another Tn5 be used (e.g. purchased from other kits), modification of Tn5 dilution will be essential.
(iv) PCR amplification, adapter extension and barcoding: Mix the 12 µl mixture from (iii) with 5 µl 5× Q5 reaction buffer, 1 µl 10 mM dNTP, 1 µl each of 5 µM Nextera i5 and i7 primer, 0.25 µl (0.5 units) Q5 High-Fidelity DNA polymerase and 19.75 µl H_2_O.
(v) Thermocycling program: 72 °C for 4 min, 98 °C for 30 s, followed by 15–18 cycles (15–16 for adults, 17–18 for larvae) of [denaturation (98 °C for 15 s), annealing (67 °C for 20 s), extension (72 °C for 90 s)] and then cool to 4 °C.
(vi) Size selection with beads (300–600 bp): Mix the 40 µl PCR product from (iv) with 22 µl beads. Incubate the mixture for 10 min at RT and then for 5 min on a magnetic stand. Transfer the 62 µl supernatant to a new tube and mix it with 10 µl of fresh beads. Incubate the mixture for 10 min at RT and then again for 5 min on a magnetic stand. Remove the supernatant and wash 2 times with 200 µl 80% ethanol while keeping the tube on the magnetic stand. Remove all the ethanol by pipetting and let beads dry for a few minutes. Remove the tube from the stand and resuspend beads in 12 µl 10 mM Tris-HCl (pH = 8.0) and incubate for 5 min. Place the tube back on the magnetic stand and incubate for 5 min. Transfer the 12 µl supernatant (library) to a new tube without disturbing the beads.
(vii) Quantification and sequencing: The library (300–600 bp fragments of PCR products from (v), or desired size) can be examined either by agarose gel or on an Agilent 2100 Bioanalyzer, and then sequenced on Illumina instruments.

We have sequenced several genomic libraries prepared as described above of individual free-living adults and iL3s of *S. stercoralis* preserved in water or ethanol. When we mapped the raw reads to the reference genome (PRJEB528.WBPS11) with bwa [47] we noticed that the percentage of reads that did map to the reference as determined with Qualimap [48] was rather variable. The mapping ratios for the individuals included in our recent publication [35] (GenBank: PRJNA517237) ranged between 32.3 and 95.2% for adults (mean = 79.6%, median = 92.7%, *n* = 10) and between 3.5 and 58.4% for infective larvae (mean = 21.1%, median = 13.0%, *n* = 26). For comparison, we also examined the libraries of individual *S. stercoralis* in our earlier publication [26] (GenBank: PRJEB20999), for which a commercial low input kit not relying on Tn5 tagmentation had been used to prepare the genomic libraries. The mapping ratios for adults (no iL3s were sequenced for this paper) ranged between 28.3 and 81.24% (mean = 55.9%, median = 60.1%, *n* = 23).

While even the lowest proportion of mapped reads (3.5%) for the individual iL3 still provided us with sufficient sequence information for phylogenetic analysis, the variability we observed illustrates that there is room for methodological improvement. A higher mapping ratio will increase the number of samples that can be multiplex-sequenced per lane and thus further reduce the cost. We randomly looked at some of the un-mapped reads and most of them did not map to any bacterial genome or the host genome, indicating that the majority of un-mapped reads were not derived from bacterial or host tissue contamination. Many un-mappable reads are probably the product of artefacts associated with the very low DNA input, such as PCR-induced errors in the process of the increased number of amplification cycles or overtagmentation of the DNA due to an excess of Tn5 [46].

## Conclusions

We do appreciate that for many people whole genome sequencing is not a primary interest and may also not be within the limits for what the funds had been approved for. Also, it requires a rather sophisticated and time-consuming analysis. Therefore, we would like to stress that *18S* and *cox*1 sequence information is most valuable in order to put a local *S. stercoralis* population into a global context. Above this, if specimens are preserved and stored properly, whole genome analysis can be performed later for selected, particularly interesting samples. In many areas where the conditions appear favorable for *S. stercoralis* transmission, no or very few studies have been conducted (e.g. for more than half of the African countries we could not find any *S. stercoralis* prevalence data in the published literature). Among the rather few available studies (e.g. case reports, clinical diagnosis, community surveys) most were solely based on morphology and did not provide any molecular information. Therefore, we hope this methodological article will encourage people around the world who encounter *S. stercoralis* in the process of their work to participate in a community effort to clarify the genetic structure, phylogenetic relationships and taxonomic status of *S. stercoralis* populations in different hosts and geographical locations.

## Data Availability

Not applicable.

## Abbreviations

DMF: N,N-dimethylformamide
HVR: hyper-variable region
iL3: infective third-stage larva
NTD: neglected tropical disease
*SSU*: small subunit (*18S*) ribosomal DNA
STH: soil-transmitted helminthiasis
TDE: tagment DNA enzyme
